# Soft tissue microcirculation around the healthy Achilles tendon: a cross-sectional study focusing on the Achilles tendon and dorsal surgical approaches to the hindfoot

**DOI:** 10.1186/s13018-018-0850-x

**Published:** 2018-06-07

**Authors:** Kajetan Klos, Boyko Gueorguiev, John Bennet Carow, Ali Modabber, Sven Nebelung, Bong-Sung Kim, Klemens Horst, Christian David Weber, Matthias Knobe

**Affiliations:** 1 Department of Foot and Ankle Surgery, Catholic Hospital Mainz, Mainz, Germany; 2 AO Research Institute Davos, Davos, Switzerland; 3 Department of Orthopaedic Trauma, University of Aachen Medical Center, Aachen, Germany; 4 Department of Oral and Maxillofacial Surgery, University of Aachen Medical Center, Aachen, Germany; 5 Department of Radiology, University of Aachen Medical Center, Aachen, Germany; 6 Department of Plastic Surgery, Reconstructive and Hand Surgery, University of Aachen Medical Center, Aachen, Germany

**Keywords:** Achilles tendon, Doppler/white light spectroscopy, Hindfoot surgical approach, Humans, Microcirculation

## Abstract

**Background:**

Dorsal approaches to the hindfoot are frequently used. Furthermore, the vascular supply is discussed as a possible cause for ruptures and degeneration of the Achilles tendon. The aim of this study was to evaluate the microperfusion of three possible posterior approaches to the hindfoot and different areas of the Achilles tendon.

**Methods:**

In 111 subjects, a laser Doppler/white light spectroscopy was used to measure microperfusion in terms of blood flow (Flow) and capillary venous oxygen saturation (SO2) in the hindfoot and Achilles tendon. Measurements were performed at two measurement points (MP, proximal and distal) of three dorsal approaches (medial, lateral and central) and inside the Achilles tendon.

**Results:**

Microperfusion differed partially between the surgical approaches. The medial and the lateral approaches were significantly superior to the central approach with regard to Flow in both MP (*p* <  0.001), while SO2 was significantly higher at the proximal measurement point (MP 1; *p* <  0.001). In this area, the lateral approach was significantly superior to the medial approach regarding Flow (MP 1; *p* = 0.012).

The Achilles tendon exhibited a significantly reduced microperfusion 5 cm proximal to the calcaneal tubercle (SO2 *p* = 0.001; Flow *p* = 0.048). Demographic factors, such as body mass index and age, had different effects. Microcirculation was partially superior in men and negatively affected by smoking.

**Conclusions:**

Soft tissue microcirculation on the lateral and medial side of the healthy Achilles tendon was better than centrally on the tendon. Proximally, the lateral approach was better than the medial approach. These circumstances could provide advantages regarding the surgical approach. The Achilles tendon exhibited significantly reduced microperfusion at the typical side of degeneration and rupture. This circumstance could be a possible cause of degenerative processes.

## Background

Dorsal surgical approaches to the hindfoot are frequently used for a variety of reasons, such as the treatment of Achilles tendon ruptures, tendinopathies, and Haglund’s deformity [1–8]. Achilles tendon lengthening may also be necessary in the context of pes planovalgus [9] corrections and for treatment of Charcot arthropathies [10] or other deformities [11]. Furthermore, hindfoot arthrodesis and osteosynthesis can be treated via a dorsal approach [12]. However, wound healing problems are not uncommon in this region. In a report by Paavola et al. [2], a series of patients were operated because of the presence of chronic Achilles tendon degeneration. Of these 423 patients, 14 developed skin necrosis and 11 developed superficial infections. This data is confirmed by other studies [1].

Three surgical approaches to the dorsal hindfoot are generally used: central, medial, or lateral to the Achilles tendon [3–5]. The lateral approach provides good visualization of the posterior distal upper and lower ankle and is therefore well suited for hindfoot arthrodesis and fracture repair [6–8]. However, due to the proximity of the sural nerve, there is a risk of nerve injury accompanied with this approach. The central approach to the Achilles tendon is particularly suitable for treatment of ruptures and tendinopathies [1, 13]. According to Hammit et al. [14], a central approach with subsequent longitudinal incision straight through the Achilles tendon provides excellent visualization of the distal tibia, ankle, and hindfoot and can also be used for osteosyntheses, arthrodeses, and osteotomies. The main disadvantage of the central approach, however, is the possible resulting inflammation or painful scarring [14]. A medial approach is favored by many surgeons as it avoids scarring on the tendon and injury to the sural nerve [3, 5, 15]. Ruptures and degenerations typically occur in the middle of the tendon, and the vascular supply may be a contributing factor [16]. The interdependence between the vascular supply and tendon pathologies, however, is controversially discussed, since different studies have come to opposing conclusions [17, 18].

In the current study, comparison was made for soft tissue microperfusion of the healthy hindfoot at different areas of the Achilles tendon between the three posterior surgical approaches to draw conclusions about the wound healing potential and to evaluate microperfusion in the tendon itself. In addition, the effects of various demographic factors, such as body mass index (BMI), smoking, gender, and age, were evaluated.

## Methods

A noninvasive measurement of the microperfusion of the hindfoot and Achilles tendon was performed in 111 volunteer participants with the use of an O2C device (oxygen to see, laser Doppler/white light spectroscopy, LEA Medical Technology, Gießen, Germany). Out of the 111 subjects, 65 were men and 24 were smokers. Further characteristics are provided in Table 1. All participants provided written informed consent to participate in the study and have their data published. Exclusion criteria were peripheral arteriovenous obstructive disease, insulin-dependent diabetes mellitus, macroangiopathy, and previous surgeries or injury in the measurement area.

**Table 1:** Subject characteristics

Characteristic	Mean	SD	Range
Age (years)	28.9	10.2	18.9–66.7
BMI (kg/m^2^)	22.3	2.5	17.9–30
Systolic blood pressure (mmHg)	111	37	103–145
Diastolic blood pressure (mmHg)	79	5	67–90

*BMI* body mass index

**Fig. 1:**
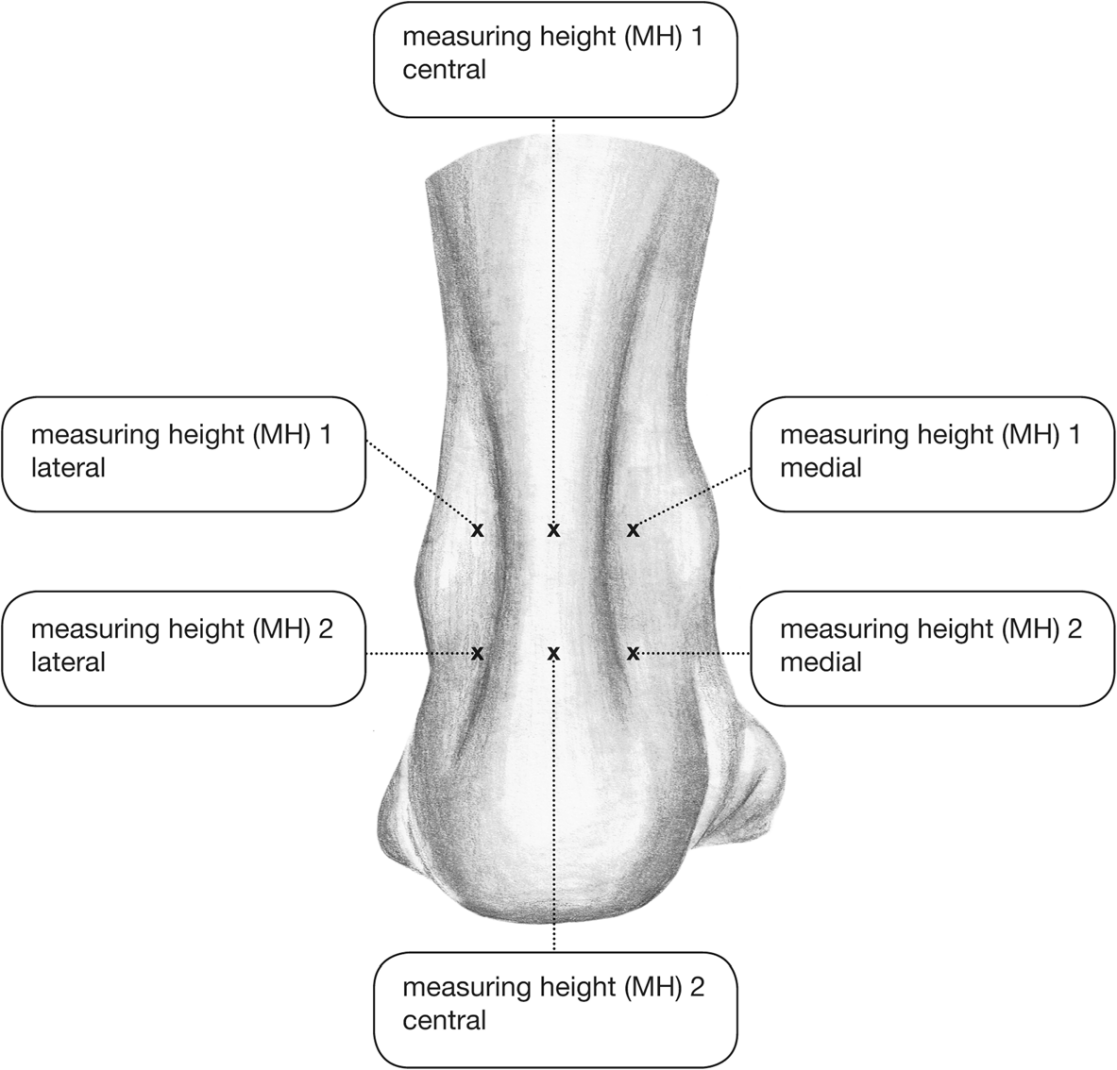
The six different measurement points. At the central points, the microperfusion was measured in 2- and 8-mm depth, whereas at the lateral and medial points it was measured in 2-mm depth only

Measurements were carried out with each subject in a resting prone position. Firstly, a 2-mm-depth (superficial) flat probe was used to define six measuring points. The measurements were obtained for each subject at medial, lateral, and central (on the tendon) sites at 5 cm (measuring height MH1) and at 1 cm (measuring height MH2) proximal to the calcaneal tubercle (Fig. 1). The following two hemodynamic parameters were measured: blood flow (Flow, in arbitrary units [AU]) and capillary venous oxygen saturation (SO2 in %) in vessels up to 100 μm in diameter. Secondly, the microperfusion in the tendon (central) was measured with a second 8-mm-depth (in-depth) flat probe at the same locations MH1 (5 cm) and MH2 (1 cm). The parameters were compared among all subjects, as well as within several subgroups (men, women, smokers, nonsmokers).

### Statistics

Statistical analysis was performed using SPSS 21 software (IBM, Armonk, NY, USA). Descriptive data are presented as mean values ± standard error of the mean/standard deviation and range. As the Shapiro-Wilk test indicated abnormally distributed values, nonparametric tests (Spearman rank, Mann-Whitney U test, Kruskal-Wallis test) were applied. *p* value < 0.05 was considered statistically significant with a 95% confidence interval.

## Results

**Fig. 2:**
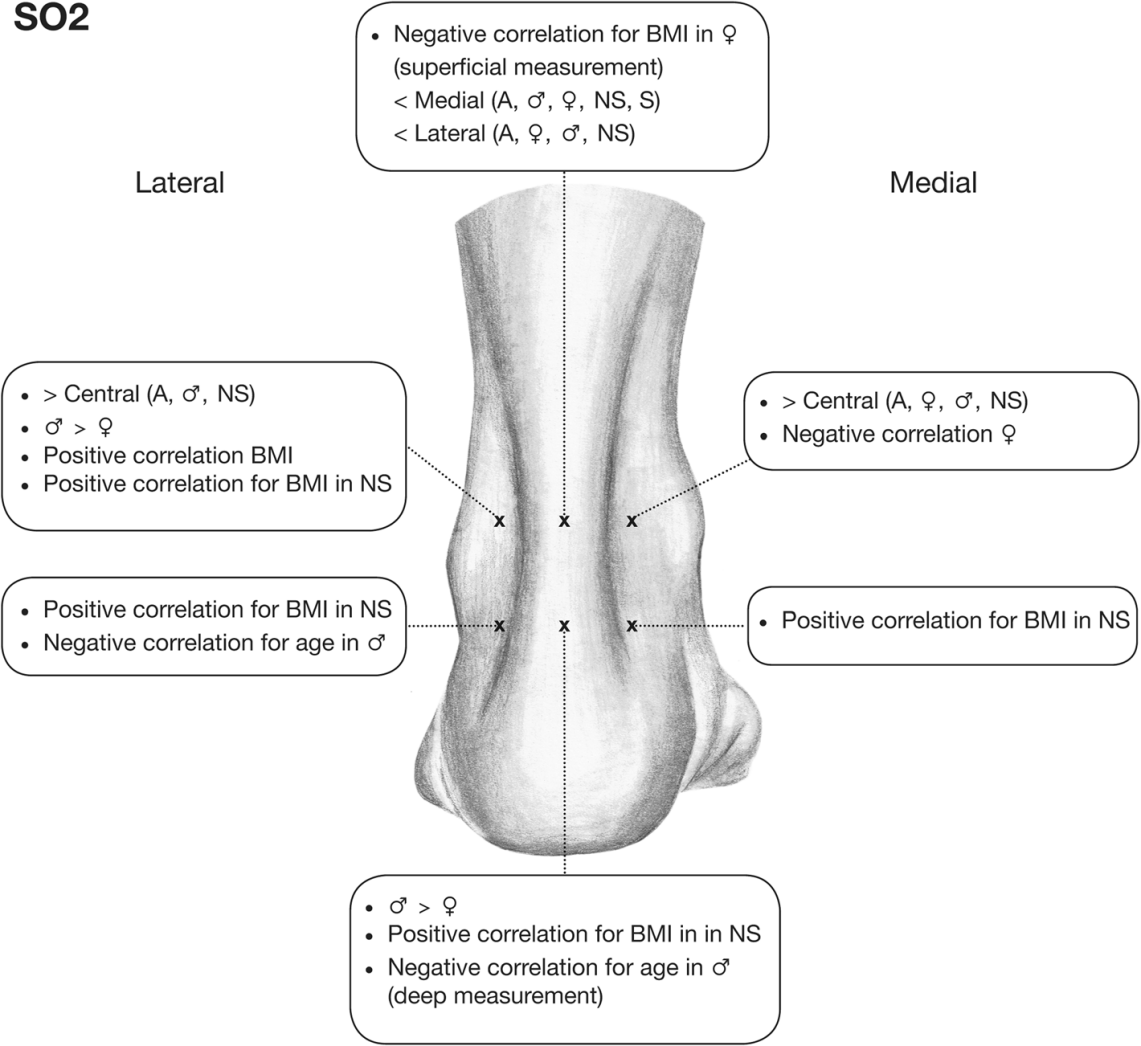
Significant correlations between SO2 and the evaluated demographic factors as well as significant differences with regard to SO2 between all measurement points are illustrated for each measurement point. (A: all; NS: nonsmokers; S: smokers)

**Fig. 3:**
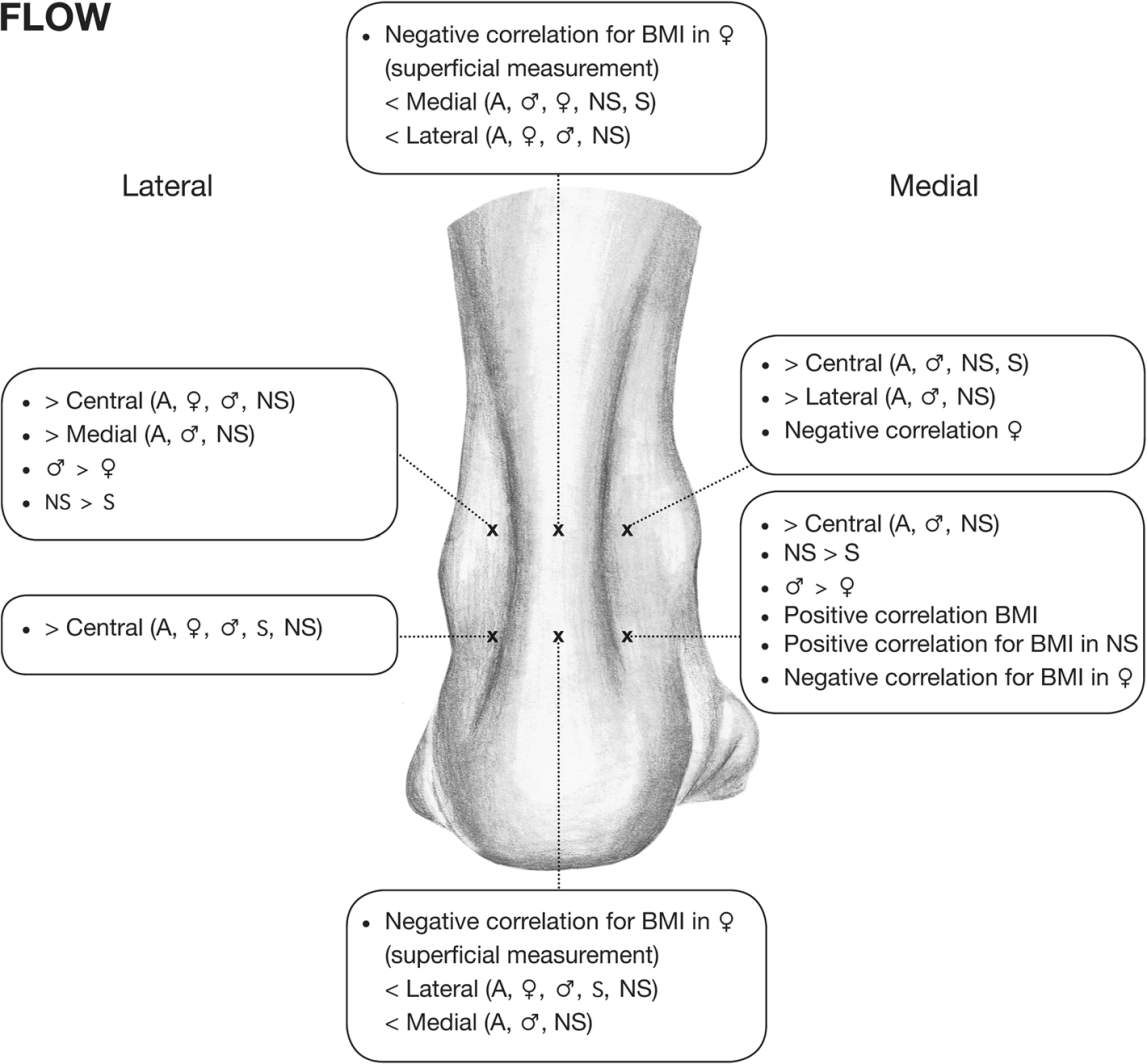
Significant correlations between Flow and the evaluated demographic factors and significant differences with regard to the Flow between all different measurement points are illustrated for each measurement point

Significant correlations between the measured parameters and evaluated demographic factors, as well as significant differences between the measurement points, are visualized in Figs. 2 and 3.

### Comparison of dermal microperfusion centrally against lateral and medial

#### Measuring height MH1

Dermal microperfusion (SO2 and Flow) at measuring height 1 (MH1, 5 cm proximal to the calcaneal tubercle) was lower at the central superficial site (on the tendon) than at the lateral and medial superficial sites (Table 2). The differences in SO2 and Flow were significant for the entire patient population and in the men and nonsmoker subgroups. For women, there was no significant difference in SO2 between lateral and central sites (*p* = 0.188), whereas for smokers no significant differences were found in Flow between lateral and central sites (*p* = 0.144) as well as in SO2 for the central versus both lateral and medial sites (*p* = 0.092; *p* = 0.280) (Table 2).

**Table 2:** Data for measurement height MH1

Groups	Variable	Mean ± SEM				*p* value		
		cs	cd	ls	ms	cs vs. ls	cs vs. ms	ls vs. ms
All	SO2	23.5 ± 1.6	54.9 ± 2.3	38.1 ± 1.8	36.8 ± 1.9	*< 0.001*	*< 0.001*	0.751
	Flow	7.4 ± 0.6	72.4 ± 2.8	15.7 ± 0.9	13.1 ± 0.9	*< 0.001*	*< 0.001*	*0.012*
Men	SO2	23.3 ± 2.2	57.0 ± 3.0	41.9 ± 2.2	38.7 ± 2.5	*< 0.001*	*< 0.001*	0.371
	Flow	7.4 ± 0.8	77.0 ± 4.4	17.0 ± 1.1	13.1 ± 0.9	*< 0.001*	*< 0.001*	*0.008*
Women	SO2	23.8 ± 2.5	51.8 ± 3.6	32.5 ± 3.0	34.0 ± 3.0	0.188	*0.036*	0.620
	Flow	7.4 ± 0.7	65.8 ± 2.4	13.6 ± 1.3	13.2 ± 1.9	*< 0.001*	*0.004*	0.349
Nonsmokers	SO2	23.6 ± 1.8	53.6 ± 2.7	38.5 ± 2.0	37.3 ± 2.2	*< 0.001*	*< 0.001*	0.830
	Flow	7.5 ± 0.7	73.0 ± 3.1	16.6 ± 1.0	12.9 ± 1.1	*< 0.001*	*< 0.001*	*< 0.001*
Smokers	SO2	23.0 ± 3.9	59.3 ± 4.1	36.7 ± 4.4	34.9 ± 4.3	0.092	0.280	0.641
	Flow	7.1 ± 1.0	70.1 ± 6.3	12.1 ± 1.6	14.1 ± 1.8	0.144	*0.004*	0.400

SO2 in %, Flow in AU

*cs* central superficial (2 mm deep), *cd* central in-depth (8 mm deep), *ls* lateral superficial (2 mm deep), *ms* superficial (2 mm deep), *SEM* standard error of the mean

#### Measuring height MH2

At measuring height 2 (MH2, 1 cm proximal to the calcaneal tubercle), the Flow was significantly higher at the lateral and medial superficial sites than at the central superficial site (on the tendon) among all subjects, and also for men and nonsmokers (Table 3). In women and smokers, a significant difference was detected in Flow only between the lateral and central superficial sites (*p* = 0.012). SO2 did not differ significantly among the three sites.

**Table 3:** Data for measurement height MH2

Groups	Variable	Mean ± SEM				*p* value		
		cs	cd	ls	ms	cs vs. ls	cs vs. ms	ls vs. ms
All	SO2	36.0 ± 2.0	70.1 ± 1.4	36.0 ± 2.0	41.0 ± 1.9	0.741	0.232	0.176
	Flow	12.4 ± 1.7	84.4 ± 4.6	20.2 ± 1.4	17.7 ± 1.1	*< 0.001*	*< 0.001*	0.259
Men	SO2	36.3 ± 2.8	74.0 ± 1.4	37.5 ± 2.5	43.1 ± 2.4	0.880	0.384	0.268
	Flow	13.5 ± 2.6	89.9 ± 6.2	21.5 ± 2.0	20.5 ± 1.6	*< 0.001*	*< 0.001*	0.769
Women	SO2	35.5 ± 3.0	64.6 ± 2.6	33.8 ± 3.2	38.1 ± 3.0	0.697	0.253	0.329
	Flow	10.89 ± 1.8	76.5 ± 6.6	18.0 ± 1.6	13.7 ± 1.4	*< 0.001*	0.180	0.082
Nonsmokers	SO2	36.3 ± 2.4	69.5 ± 1.7	35.8 ± 2.2	41.2 ± 2.0	0.914	0.320	0.292
	Flow	12.9 ± 2.0	86.3 ± 5.5	20.4 ± 1.6	18.6 ± 1.3	*< 0.001*	*< 0.001*	0.538
Smokers	SO2	34.8 ± 4.0	72.2 ± 2.4	37.0 ± 4.7	40.4 ± 5.1	0.526	0.466	0.366
	Flow	10.7 ± 2.8	77.4 ± 7.7	19.3 ± 2.7	14.2 ± 2.0	*0.012*	0.344	0.258

SO2 in %, Flow in AU

*cs* central superficial (2 mm deep), *cd* central in-depth (8 mm deep), *ls* lateral superficial (2 mm deep), *ms* superficial (2 mm deep), *SEM* standard error of the mean

### Dermal microperfusion at the lateral site compared with the medial site

#### Measuring height MH1

Comparison between the lateral and medial superficial sites of the tendon at MH1 revealed that microperfusion (Flow) was greater at the lateral site than at the medial site in the total subject population (mean Flow 15.7 AU vs. 13.1 AU; *p* = 0.012) (Table 2). In the subgroups, the difference in Flow remained significant for men (*p* = 0.008) and nonsmokers (*p* <  0.001), but not in women (*p* = 0.349) and smokers (*p* = 0.4). There were no differences regarding oxygen saturation.

### Measuring height MH2

Comparisons in Flow and SO2 between lateral and medial sites at MH2 yielded no significant differences for the subjects (Table 3).

### Microperfusion in the Achilles tendon

Measurements in the tendon (central in 8-mm depth) showed significantly lower values for MH1 versus MH2 within all subjects for SO2 (*p* = 0.001) and Flow (*p* = 0.048) (Tables 2, 3, and 4). In the subgroups, those differences remained significant for SO2, but not for Flow (Tables 2, 3, and 4).

**Table 4:** Comparison between MH1 and MH2 for SO2 and Flow in the Achilles tendon

Groups	Variable	*p* value
Total	SO2	*< 0.001*
	Flow	*0.048*
Men	SO2	*< 0.001*
	Flow	0.056
Women	SO2	*< 0.001*
	Flow	0.356
Nonsmokers	SO2	*< 0.001*
	Flow	0.054
Smokers	SO2	*0.001*
	Flow	0.568

### Effect of BMI

Within all subjects, BMI correlated significantly and positively with lateral (superficial) SO2 at MH1 (*p* = 0.011) and with medial (superficial) Flow at MH2 (*p* = 0.010) (Figs. 2 and 3).

### Effect of age

Some isolated significant negative correlations with age were detected at MH2 for men regarding SO2 in the tendon (in-depth central, *p* = 0.037) and for lateral (superficial) SO2 (*p* = 0.038) (Fig. 2).

### Effect of gender

Comparison between the genders among all subjects revealed significantly higher values for men at MH1 for SO2 (*p* = 0.013) and Flow at the lateral site (*p* = 0.041), as well as at MH2 for SO2 in the tendon (in-depth; *p* = 0.002) and for medial Flow (*p* = 0.001, Figs. 2 and 3).

### Effect of smoking

Comparison between nonsmokers and smokers among all subjects revealed a significantly better microperfusion for nonsmokers versus smokers in terms of Flow at MH1 lateral (superficially, *p* = 0.014) and at MH2 medial (superficially, *p* = 0.043) (Fig. 3).

## Discussion

The O2C device as a tool for noninvasive detection of hemoglobin saturation and blood flow using laser Doppler technique and white light spectroscopy allows for valid and reproducible quantitative determination of microcirculatory parameters [19–24], as well as identification of possible influencing factors in healthy subjects [23, 25]. Currently, O2C studies have provided evidence for altered soft tissue microcirculation after peripheral artery occlusive disease [26] as well as for individual healing tendencies depending on the microcirculatory parameters in patients with burns [27, 28] and chronic diabetic foot ulcers [19]. In this context, recent data in the treatment of fractures of the thoracolumbar spine indicated differences in microcirculation between open and minimally invasive approaches [24]. High oxygen saturation and high blood flow values clearly contribute to a good prognosis of wound healing. To the best of our knowledge, there are no existing studies investigating microcirculation as a parameter for evaluation of possible surgical approaches at the dorsal hindfoot.

The lateral approach is supplied by the peroneal artery. This approach exhibited the best microcirculation in the current study with a significant difference compared to the central approach for Flow, partly for SO2, and compared to the medial approach to some extent for Flow. Surgical access to the Achilles tendon through this site is considered as a critical intervention because of its proximity to the sural nerve [29], even though it provides advantages for removing Haglund exostoses because of the position of the calcaneus in relation to the Achilles tendon. Little et al. reported good results with low complication rates for treating distal tibial and talar fractures using this lateral approach [7]. An anatomical study evaluating the lateral approach for hindfoot arthrodesis demonstrated a risk for injuries of the venous system [6]. In addition, the lateral malleolar branches of the peroneal artery were often endangered while the sural nerve remained intact in all specimens that were evaluated in this study [6].

In the present study, the lowest microperfusion was observed centrally (superficially) on the Achilles tendon. This finding is consistent with previous studies of lower limb angiosomes [30]. Taylor and Pan [30] showed that the two angiosomes of the peroneal vessels and the vessels from the posterior tibial artery meet on the lower leg in the midline above the Achilles tendon. In a clinical study, Hammit et al. [14] reported that an approach direct over the Achilles tendon yielded good results and low complication rates. However, the authors rated the development of a painful or unsightly scar as critical, although no such a complication occurred in their study [14]. They speculated that their good results were due to the access in the watershed area between the two angiosomes [14].

The medial approach, supplied by the posterior tibial artery, exhibited significantly better microcirculation than the central approach in the present study except of the SO2 for the distal measurement point, but was somewhat inferior to that of the lateral area (MH1 Flow). The medial approach is widely used to treat Haglund exostoses and Achilles tendon ruptures, because here the sural nerve cannot be damaged and the scar is not as problematic as on the tendon [3, 5, 15].

The measurements superficially and deep in the tendon showed significantly worse microperfusion at MH1 among all subjects. This corresponds with the experience that ruptures and degenerations typically occur at this level and contradicts the anatomical study of Schmidt-Rohlfing et al. [17] who could not see any differences in the middle part of the tendon. However, our results confirmed the anatomical study of Chan et al. [18], who did a histological and angiographic study and concluded that the middle Achilles tendon is hypovascular [18].

Higher BMI is considered a risk factor for Achilles tendon degeneration [16]. Thus, it was expected that perfusion would also correlate negatively with BMI. A negative correlation between perfusion in the tendon and BMI was not seen in this study. On the contrary, within all subjects, BMI correlated significantly and positively with lateral SO2 at MH1 and with medial Flow at MH2. However, BMI negatively correlated with superficial perfusion of the tendon, however, only for smokers and women.

A negative correlation between age and tendinous blood flow was detected for men, which is consistent with previous observations that female gender is a protective factor for Achilles tendon rupture [31] and may also allude the high incidence of Achilles tendon rupture for men in the fourth and fifth decades of their life [32]. These findings may be caused by an increased vascular stiffness, decreased vascular density, or impaired vascular organization, which can lead to a decreased blood supply in aging skin [33]. For that reason, the results of operative treatment of geriatric foot and ankle fractures are essentially determined by the soft tissue management [12] and minimally invasive techniques are recommended in elderly patients [34]. There was also an improved perfusion in nonsmokers compared to smokers seen for SO2 lateral at MH 1 and for Flow medial at MH 2. In a large meta-analysis of 140 studies and 479,150 patients across all surgical specialties, smokers were found to have a higher incidence of postoperative healing complications compared to nonsmokers [35]. In contrast to this, a meta-analysis in trauma surgery, and treatment of closed calcaneal fracture specifically, involving 1559 patients with 1651 fractures, smoking was not found to have a significant influence on posttraumatic and postoperative wound complications [36].

In line with the study of Gardner et al. [37], our data showed a gender difference in microcirculatory parameters in favor of males. This could be caused by gender-specific physiological differences, such as the ratio of skeletal muscle and thickness of subcutaneous fat [38]. Thicker muscle, less subcutaneous fat, and higher blood vessel density in men could lead to this difference in microcirculation, even in rest. However, we cannot estimate microcirculation in a trauma situation, which can be different. Further studies are necessary to clarify the question of gender-specific influence on microcirculatory parameters, since some studies could not show any dependence from gender [23].

A study by Bruggeman et al. [39] examined 164 patients with Achilles tendon repair. More than 10% of the patients experienced wound healing problems [39]. Among others, smoking and female gender were identified as risk factors, whereas BMI and age did not play a considerable role [39]. In the current study, we confirmed the negative influence of smoking. Women also exhibited reduced perfusion compared to men. We found both negative and positive correlations between BMI and superficial dermal perfusion. Thus, the results are consistent with those of Bruggeman et al. [39].

This examination was carried out exclusively on healthy and uninjured subjects. Conclusions on patients suffering from a degenerative hindfoot disease or hindfoot injury are therefore limited. Although the study confirms the presumed superficial “underperfusion” of the tendon in the centered area, a change in perfusion as the sole cause of Achilles tendon degeneration is not substantiated by limited data. Based on this work, the lateral approach may be recommended but the extent to which microcirculation is affected by the surgical approach itself cannot be answered. Dragu et al. [40] showed significant changes in the microperfusion parameters of the evaluated areas after abdominoplasty. These authors showed that previously well-oxygenated parts of the approach underwent a significant decrease in oxygen saturation upon mobilization and suturing, while other parts of the wound edges showed an increase of microperfusion [40].

Thus, it is possible that a cut next to the watershed area above the Achilles tendon may lead to a reduced blood flow from this direction (for the medial approach from lateral and vice versa) as there are likely very few collaterals in this area and that mobilization of the tissue from the medial or lateral side may further affect microperfusion. This consideration may support a central access route to the Achilles tendon as this area is supplied from both the posterior tibial artery and the peroneal artery as it is suspected by Hammit et al. [14]. The disadvantage of this approach is that it may lead to a painful or unsightly scar.

Based on the determination of the microcirculation in healthy subjects, a final assessment of the different approaches is not possible, and further studies are necessary applying laser Doppler/white light spectroscopy in investigating the perfusion of the different surgical approaches to the hindfoot during and after surgery.

## Conclusion

Dermal microcirculation is significantly limited above the Achilles tendon. Microcirculation is better lateral and medial than central on the tendon. It is also better on the proximal lateral side than proximal medial. Furthermore, differences in microperfusion vary between men and women with a superiority for men having higher values. The negative effect of smoking on microcirculation was partially confirmed. The Achilles tendon shows a significantly reduced microperfusion 5 cm proximal to the calcaneal tubercle. The influence of these results on everyday clinical practice requires further clarification in additional investigations, including clinical studies.

## Abbreviations

BMI: Body mass index
Flow: Blood flow (arbitrary unit)
MH: Measuring height
NS: Nonsmokers
O2C: Oxygen to see, laser Doppler/white light spectroscopy
S: Smokers
SO2: Capillary venous oxygen saturation (percent)
